# How Much Does Malaria Vector Control Quality Matter: The Epidemiological Impact of Holed Nets and Inadequate Indoor Residual Spraying

**DOI:** 10.1371/journal.pone.0019205

**Published:** 2011-04-29

**Authors:** Andrea M. Rehman, Mike Coleman, Christopher Schwabe, Giovanna Baltazar, Abrahan Matias, Irina Roncon Gomes, Lee Yellott, Cynthia Aragon, Gloria Nseng Nchama, Themba Mzilahowa, Mark Rowland, Immo Kleinschmidt

**Affiliations:** 1 MRC Tropical Epidemiology Group, London School of Hygiene and Tropical Medicine, London, United Kingdom; 2 Liverpool School of Tropical Medicine, Liverpool, United Kingdom; 3 Medical Care Development International, Silver Spring, Maryland, United States of America; 4 Medical Care Development International, Malabo, Equatorial Guinea; 5 Medical Care Development International, Bata, Equatorial Guinea; 6 Ministry of Health and Social Welfare, Malabo, Equatorial Guinea; 7 Malaria Alert Centre, Malawi College of Medicine, Blantyre, Malawi; 8 Department of Disease Control, London School of Hygiene and Tropical Medicine, London, United Kingdom; Menzies School of Health Research, Australia

## Abstract

**Background:**

Insecticide treated nets (ITN) and indoor residual spraying (IRS) are the two pillars of malaria vector control in Africa, but both interventions are beset by quality and coverage concerns. Data from three control programs were used to investigate the impact of: 1) the physical deterioration of ITNs, and 2) inadequate IRS spray coverage, on their respective protective effectiveness.

**Methods:**

Malaria indicator surveys were carried out in 2009 and 2010 in Bioko Island, mainland Equatorial Guinea and Malawi to monitor infection with *P.falciparum* in children, mosquito net use, net condition and spray status of houses. Nets were classified by their condition. The association between infection and quality and coverage of interventions was investigated.

**Results:**

There was reduced odds of infection with *P.falciparum* in children sleeping under ITNs that were intact (Odds ratio (OR): 0.65, 95% CI: 0.55–0.77 and OR: 0.81, 95% CI: 0.56–1.18 in Equatorial Guinea and in Malawi respectively), but the protective effect became less with increasingly worse condition of the net. There was evidence for a linear trend in infection per category increase in deterioration of nets. In Equatorial Guinea IRS offered protection to those in sprayed and unsprayed houses alike when neighbourhood spray coverage was high (≥80%) compared to those living in areas of low IRS coverage (<20%), regardless of whether the house they lived in was sprayed or not (adjusted OR = 0.54, 95% CI 0.33–0.89). ITNs provided only personal protection, offering no protection to non users. Although similar effects were seen in Malawi, the evidence was much weaker than in Equatorial Guinea.

**Conclusions:**

Universal coverage strategies should consider policies for repair and replacement of holed nets and promote the care of nets by their owners. IRS programs should ensure high spray coverage since inadequate coverage gives little or no protection at all.

## Introduction

Insecticide treated nets (ITN) and indoor residual spraying (IRS) are the two pillars of malaria vector control in Africa. Both methods have been proven to be effective in reducing the risk of infection with malarial parasites, clinical disease and child mortality [1], [2], [3], [4], [5]. Both methods have known limitations. Pyrethroids are currently the only class of insecticide that can be used on ITNs and it is not clear to what extent this compromises their effectiveness against pyrethroid resistant mosquitoes, particularly once they have acquired holes through wear and tear and therefore no longer provide a complete physical barrier. For IRS programmes a wider choice of insecticides is available, at least in principle, but in areas with long or multiple seasons or perennial malaria transmission it is difficult to ensure lethal residual insecticide on walls at all times. This may necessitate multiple spray rounds which can make it more difficult to achieve and maintain high spray coverage.

In this study we have used observational data from Equatorial Guinea and Malawi to investigate the potential impact of two challenges with which malaria vector control programmes are confronted: 1) do holes in mosquito nets reduce their effectiveness in preventing malarial infections in children; and 2) does IRS provide protection against infection if spray coverage is low or moderate?

Untreated nets are known to provide some protection against malaria when in good condition but once they become holed this protection is lost [6], [7]. Treatment of holed nets with pyrethroid insecticide restores the protective effect [8]. This situation may change in some areas of pyrethroid resistance. Experimental hut studies have shown that in south Benin pyrethroid resistant *An.gambiae* were able to blood feed on persons sleeping under holed insecticide treated nets whilst similarly holed nets did inhibit blood feeding in north Benin where vectors were susceptible [9]. In Tanzania, insecticide treated nets were still effective after several years use provided they remained intact [10]. There have been no reports of epidemiological impact and loss of protective effect in people resulting from holes in ITNs. We used malaria indicator survey (MIS) data from 2009 and 2010 from Bioko Island, from continental Equatorial Guinea and from Malawi, to determine how the risk of infection with *P.falciparum* differed between children sleeping under no nets, sleeping under untreated but physically intact nets, sleeping under treated nets with holes, and sleeping under treated nets with no holes, after adjusting for confounders.

It is generally recommended that IRS should be provided at high coverage to give its full protective effect as a vector control tool [11]. We used the same MIS data from Bioko Island, mainland Equatorial Guinea and Malawi referred to above to investigate the impact of varying neighbourhood spray coverage levels on individual risk of infection after adjusting for confounders including ITN usage and whether the house in which the individual slept had been sprayed or not.

## Methods

### Interventions and malaria indicator surveys

#### 1. Bioko Island

Bioko is the main island of Equatorial Guinea, situated approximately 30 miles off the coast of Cameroon, with a population of about 200,000. Malaria has historically been hyperendemic in Bioko with year round transmission. Annual entomological inoculation rates (EIR) of over 250 and 750 infectious bites per person per year by *An.gambiae* and *An.funestus* respectively were recorded before intensive malaria control was introduced [12]. The Bioko Island Malaria Control Project (BIMCP) was launched in 2004 in collaboration with the government of Equatorial Guinea. IRS was introduced in February 2004 using the pyrethroid deltamethrin (K-othrine WDG, Bayer Crop Science, Isando, South Africa), followed by biannual spraying with the carbamate bendiocarb (Ficam™, Bayer) from 2005. Strengthening of diagnosis through training in microscopy and the introduction of rapid diagnostic tests (RDT), the introduction and training of health facility staff in case management using Artemisinin Combination Therapy (ACT), expanding access to Intermittent Preventative Treatment for pregnant women (IPTp), and extensive information education and communication activities were started in 2005. Mass door-to-door distribution of the long lasting insecticidal net (LLIN) PermaNet 2.0 (Vestergaard Frandsen, Lausanne, Switzerland) was carried out in November and December 2007, supplying and assisting in hanging one net per sleeping area at an average of three nets per household. Entomological surveillance was carried out continuously and Malaria indicator surveys were undertaken annually in 18 sentinel areas to evaluate the impact of the control measures as reported previously [13], [14], [15].

The second five year phase of the BIMCP began in 2009. The study we are describing is based in part on data from the first two BIMCP annual surveys (of the second five year phase) which were carried out from August to September 2009 and 2010. One spray locality (areas used to carry out house spraying) was randomly selected with probability proportional to size in each sentinel area. A fixed number of houses (400 in each urban and 100 in each rural site) were selected by systematic sampling.

#### 2. Mainland Equatorial Guinea

Mainland Equatorial Guinea has a number of malaria vectors, which have been described in detail elsewhere [16]. The main vector is *An.gambiae*. The Equatorial Guinea Malaria Control Initiative (EGMCI), which is linked to the BIMCP, initiated malaria control measures in mainland Equatorial Guinea (EG) from 2007, similar to interventions carried out in Bioko, except that every province was designated to receive either IRS or LLINs by mass distribution. Centro Sur and Wele Nzas provinces received PermaNet 2.0 (Vestergaard Frandsen) LLINs in August to September 2007 and 2008 respectively. In Litoral province houses were sprayed semi-annually from August 2007 through September 2009 with the pyrethroid insecticide Alpha Cypermethrin (Fendona™, Avima/BASF, South Africa and HI Kara, India), in January 2010 with the carbamate insecticide bendiocarb (Ficam™, Bayer, South Africa), and from July 2010 with the pyrethroid insecticide deltamethrin (K-Orthrin™, Bayer, South Africa). In Kie Ntem province IRS was carried out semi-annually with Alpha Cypermethrin from July 2008, with Deltamethrin™ from January 2010 and with bendiocarb in July 2010. In addition, villages not covered by IRS in Litoral (seven in Mbini and 33 in Cogo) received LLINs in February 2009. Other malaria control measures were similar to those in Bioko.

Programme monitoring and evaluation was also along the lines of that carried out on Bioko, including household MIS between April and June each year. In 2007 household listings were compiled in 17 sentinel sites from which households were randomly sampled each year for participation in the survey. We are using data from two MIS carried out in 2009 and 2010.

#### 3. Malawi

Malaria is endemic in Malawi with the main transmission season between October and April. The predominant malaria vectors are *An. arabiensis* and *An. funestus*.

The Malaria Decision Support System, as part of the Innovative Vector Control Consortium [17] (IVCC), conducted continuous entomological monitoring since 2007 and malaria indicator surveys in April 2009 and 2010 at 14 sentinel sites in Nkhota Khota district in Malawi. Vector control has primarily been via ITNs, with distribution campaigns in 2006 and 2008 to the most vulnerable groups. Continuous distribution of ITNs has been through antenatal clinics. In 2008, supported by the Presidents Malaria Initiative, annual IRS operations, with the pyrethroid lambda-cyhalothrin™ (ICON, Syngenta, Switzerland) began as a pilot of 25,000 households in Nkhota Khota district. In 2009 four sites (Makoka, Mazanduwa, Kalungama and Kamtekeke) received IRS and with the scale up of activities a fifth site (Ambali) was sprayed in 2009. Households for the MIS were selected from strata formed by dividing sentinel sites into quadrants from which households were systematically selected, to ensure the greatest geographical spread within the site. The data from the two household surveys were used in the study we are reporting.

### Data preparation and Statistical analysis

All data were collected using Personal Digital Assistants (PDA). Children less than 15 years of age were tested for *P.falciparum* using ICT™ Malaria Combo Cassette Test (ML02, R&R, Cape Town, South Africa) in Bioko, mainland Equatorial Guinea and in Malawi. Children under one year old were excluded from analysis *a priori* because maternal antibodies could provide protection against infection.

Adherence to malaria control measures for each child were derived from the questionnaires; whether the child slept under an ITN the night before the survey; the condition of the net and whether the house in which the child lived had been sprayed during a reference period. Nets were classified into categories based on whether they were LLIN, treated or untreated nets, whether they were intact and if holed, the size of the largest hole to generate a net condition variable (see table 1 for details). Small holes were defined as being smaller than a D sized torch battery. Big holes were defined as at least one hole that could fit a D sized torch battery. Net condition was classified by the size of the largest hole only, not by the number of holes. In a small number of nets (2% of all nets, n = 204) the condition of the net was unknown. Using an untreated net with holes was included in the category of worst condition as there was no evidence of a statistical difference between this category and those not using a net (p>0.2). In Equatorial Guinea where malaria transmission is year round and spraying is carried out every six months, IRS coverage was assessed over the reference period of six months prior to the survey, whereas in Malawi where IRS is carried out annually, IRS coverage was assessed over the reference period of 12 months prior to the survey.

**Table 1:** Number (and percentage) of bed nets reported.

	Bioko Island		Mainland EG		Malawi	
Type and condition of net	2009	2010	2009	2010	2009	2010
**Worst**						
**Untreated net with holes**	132 (5.6)	476 (19.4)	265 (11.5)	311 (19.0)	4 (0.6)	12 (1.7)
**Untreated net of unknown condition**	5 (0.2)	3 (0.1)	5 (0.2)	9 (0.5)	0 (0)	0 (0)
**Unknown type of net of unknown condition**	20 (0.8)	31 (1.3)	24 (1.0)	62 (3.8)	0 (0)	0 (0)
**Second worst**						
**LLIN with big holes**	223 (9.4)	104 (4.2)	78 (3.4)	78 (4.8)	137 (20.7)	130 (18.9)
**Pre-treated with big holes**	21 (0.9)	77 (3.1)	3 (0.1)	41 (2.5)	1 (0.2)	0 (0)
**LLIN or pre-treated net of unknown condition**	7 (0.3)	2 (0.1)	8 (0.3)	9 (0.5)	4 (0.6)	15 (2.2)
**Middle** 3						
**Untreated net with no holes**	257 (10.9)	704 (28.7)	381 (16.5)	354 (21.6)	0 (0)	8 (1.2)
**Second best**						
**LLIN with small holes** 4	432 (18.2)	164 (6.7)	163 (7.1)	185 (11.3)	217 (32.7)	325 (47.3)
**Pre-treated with small holes**	46 (1.9)	154 (6.3)	16 (0.7)	56 (3.4)	1 (0.2)	0 (0)
**Best**						
**LLIN** 1 **with no holes**	1094 (46.2)	348 (14.2)	1331 (57.6)	313 (19.1)	296 (44.6)	197 (28.7)
**Pre-treated** 2 **with no holes**	131 (5.5)	392 (16.0)	35 (1.5)	220 (13.4)	3 (0.5)	0 (0)
**All Nets**	**2368 (100)**	**2455 (100)**	**2309 (100)**	**1638 (100)**	**663 (100)**	**687 (100)**

1 Long lasting impregnated nets.

2 Soaked with insecticide within the last 12 months.

3 In Malawi there were too few in this category so it was combined into the second worst category for subsequent analysis.

4 Small holes are none that can fit a D sized torch battery.

Socio economic status (SES) was generated for each household using the first principal component score based on asset ownership and household characteristics [18], and converted to tertiles for analysis (See supporting information file S1 for further details).

Logistic regression was used to estimates risk of malarial infection of an individual in relation to whether they had slept under a net the night before the survey (as a proxy measure for net use in general), the category of condition of the net, whether the house had been sprayed in the reference period, the spray coverage of the neighbourhood (site), net usage of the neighbourhood, SES tertile, and age group. Due to differences in transmission patterns, health service provision and vector ecology between Equatorial Guinea and Malawi, separate models were fitted for the two countries. In Equatorial Guinea an indicator was used to distinguish between Bioko Island and the mainland, and in Malawi an indicator was used to distinguish between lakeside and non lakeside sites. Standard errors were adjusted to account for the survey design [19]. The primary sampling unit (PSU) was set to be the sentinel site. All analyses were carried out in the STATA 11.1 software package [20].

The Equatorial Guinea continental region and Bioko studies were approved by the ethics committees of the Ministry of Health and Social Welfare in Equatorial Guinea and the London School of Hygiene and Tropical Medicine. The Malawi study was approved by the Malawi College of Medicine Ethics Committee (COMREC). The parents or caregivers of all children who participated in the surveys were asked for informed written consent. Respondents were informed of the purpose of the survey, the procedure for obtaining information and blood samples, the risks and benefits in participation, confidentiality of information collected, and their right to refuse. Individuals who tested positive for malarial parasites were referred to a local clinic for appropriate treatment according to national treatment policy. No individuals or communities were randomly allocated to different treatments.

## Results

Most nets (5860/10120: 58%) reported in the MIS were long lasting impregnated nets (LLIN) (Table 1). Condition of the LLINs varied between locations; 1442/2374 (61%), 1644/2165 (76%) and 493/1321 (37%) of LLINs in Bioko Island, continental Equatorial Guinea and Malawi respectively were intact without holes (Table 1). Untreated nets were rare in Malawi. Ownership and use of ITNs was lowest in 2010 in Equatorial Guinea. On Bioko ITN ownership declined further over the two years of the study from a previous high of 94%% after the mass distribution in 2007 [15] to 23% in 2010 (table 2). In Malawi there was an increase in ITN ownership (from 53% to 68%) but only a modest rise in use (36% to 41%) between 2009 and 2010 (Table 2). IRS is carried out in all sites on Bioko Island and hence there is a greater percentage of households who have had IRS within the last six months compared to mainland Equatorial Guinea where only 8/17 sites received IRS (Table 2). In Malawi 4/14 sites had IRS in 2009 and 5/14 sites had IRS in 2010 so over all sites the coverage of IRS was moderately low.

**Table 2:** Prevalence of *P. falciparum* in 1–14 year olds and key data on malaria prevention activities.

	Bioko Island^3^		Mainland EG		Malawi	
	2009	2010	2009	2010	2009	2010
**Children 1–14 years (n)**	5972	7563	4630	4179	2061	2024
**Children with RDT** 1 **(n)**	5566(93%)	7114(94%)	4033(87%)	3714(89%)	1901(92%)	1632(81%)
**Prevalence of ** ***P. falc***	19%	24%	64%	75%	54%	46%
**95% CI**	(14, 27%)	(19, 30%)	(61, 68%)	(69, 79%)	(44, 64%)	(34, 58%)
**Use of ITN the previous**	28%	14%	22%	15%	36%	41%
**night 95% CI**	(23, 33%)	(12, 16%)	(15, 31%)	(11, 19%)	(28, 44%)	(34, 49%)
**Households (n)**	2245	2938	1742	1694	693	568
**Owning ITN**	46%	23%	44%	29%	53%	68%
**95% CI**	(38, 54%)	(20, 26%)	(32, 56%)	(24, 34%)	(42, 63%)	(58, 77%)
**Households sprayed in**	53%	66%	29%	28%	20%	25%
**reference period** 2 **95% CI**	(50, 57%)	(62, 71%)	(16, 48%)	(16, 44%)	(7, 43%)	(10, 49%)

1 *P. falciparum* status is available only for these children with rapid diagnostic test results.

2 Spraying activities were carried out at all 18 sites on Bioko Island, at 8/17 sites on continental Equatorial Guinea and is given as coverage in the last six months; Spraying activities in Malawi were carried out at 4/14 sites in 2009, and 5/14 sites in 2010 and given as coverage in the last 12 months.

3 In 2008 in Bioko Island the proportion of houses owning at least one ITN was 94% and the proportion of children <5 sleeping under an ITN was 72% [15].

The prevalence of *P.falciparum* was highest on mainland Equatorial Guinea (64% and 75% in 2009 and 2010 respectively) and lowest on Bioko Island (19% and 24% in 2009 and 2010 respectively, Table 2). In Malawi a modest decline in prevalence was observed from 54% to 46% (Table 2).

Most children (70% in Bioko and mainland EG and 61% in Malawi) either did not sleep under a net or slept under an untreated net with holes (Table 3). Net use by site was moderate for all sites and was not higher than 71% at any site (Table 3). Site spray coverage in Malawi was either very low, or very high, whereas in Equatorial Guinea there was a greater range of spray coverage among sites (Table 3).

**Table 3:** Characteristics of children analysed n = 26,429.

		Bioko and Mainland EG		Malawi	
Covariate	Covariate level	Number	Percentage	Number	Percentage
***P. falciparum***	Negative	12,245	54.8	1,748	42.8
	Positive	8,182	36.6	1,785	43.7
	Missing	1,917	8.6	552	13.5
**Protection from net**	Worst and no net	15,699	70.3	2,504	61.3
	Second worst	633	2.8	340	8.3
	Middle	1,163	5.2		
	Second best	1,071	4.8	730	17.9
	Best	2,638	11.8	511	12.5
	Missing	1,140	5.1		
**Age group**	1–4 years	8,568	38.4	1,632	40.0
	5–9 years	7,805	34.9	1,564	38.3
	10–14 years	5,971	26.7	889	21.8
**SES tertile**	Highest	7,126	31.9	1,715	42.0
	Middle	6,653	29.8	1,330	32.6
	Lowest	6,161	27.6	1,010	24.7
	Missing	2,404	10.8	30	0.7
**Indoor residual**	Living in unsprayed house	9,486	42.5	2,916	71.4
**Spraying**	House sprayed within the reference period^1^	9,159	41.0	1,134	27.8
	Missing	3,699	16.6	35	0.9
**Site spray coverage in**	0–9.9%	3,887	17.4	2,357	57.7
**last 6 or 12 months**	10–19.9%	0	0	456	11.2
	20–29.9%	231	1.0	0	0
	30–39.9%	343	1.5	0	0
	40–49.9%	3,722	16.7	0	0
	50–59.9%	4,734	21.2	0	0
	60–69.9%	6,288	28.1	425	10.4
	70–79.9%	2,138	9.6	0	0
	80–89.9%	1,001	4.5	427	10.5
	90–100%	0	0	420	10.3
**Site net use**	10–19.9%	1,099	4.9	139	3.4
	20–29.9%	5,724	25.6	418	10.2
	30–39.9%	6,543	29.3	1,174	28.7
	40–49.9%	4,117	18.4	1,064	26.1
	50–59.9%	3,929	17.6	552	13.5
	60–69.9%	402	1.8	738	18.1
	70–79.9%	272	1.2	0	0
	Missing	258	1.2	0	0

1 Reference period is 6 months for Equatorial Guinea and 12 months for Malawi.

Net condition was associated with infection with *P.falciparum* in both Equatorial Guinea (Table 4) and Malawi (Table 5). The adjusted odds ratios showed that compared to children sleeping under no net, or an untreated net with holes, children sleeping under an ITN with no holes (the best case) had reduced odds of infection (OR = 0.65, 95% CI: 0.55–0.77 in Equatorial Guinea; OR = 0.81, 95% CI: 0.56–1.18 in Malawi). Children sleeping under an ITN with small holes (smaller than could fit a D sized torch battery, the second best case) also had reduced odds of infection but the protective effect was less for those sleeping under ITNs with larger holes (tables 4 and 5). In Equatorial Guinea untreated nets with no holes offered some protection. There was no evidence that high coverage of nets offered protection to those not sleeping under nets.

**Table 4:** Odds ratios (95% CI) of infection with *P. falciparum* among 1–14 year olds in Equatorial Guinea.

Covariate	% infected (n)	Crude OR	95% CI	Adjusted OR	95% CI
**Protection from net** 1					
Worst and no net	40 (10,814)	1		1	
Second worst	33 (508)	0.73	(0.56–0.96)	0.95	(0.71–1.28)
Middle	37 (783)	0.85	(0.66–1.10)	0.85	(0.71–1.01)
Second best	30 (765)	0.62	(0.50–0.77)	0.65	(0.54–0.79)
Best	34 (1937)	0.77	(0.58–1.02)	0.65	(0.55–0.77)
**Site net use**					
<50% people	39 (11,922)	1		1	
≥50% people	37 (2885)	0.91	(0.44–1.88)	0.74	(0.50–1.09)
**IRS of the household**					
Unsprayed house	47 (7309)	1		1	
Sprayed within last 6 months	31 (7498)	0.5	(0.36–0.70)	0.9	(0.80–1.01)
**Site spray coverage last 6 months**					
0–19.9%	70 (2503)	1		1	
20–49.9%	38 (2650)	0.26	(0.11–0.59)	0.93	(0.63–1.38)
50–79.9%	31 (8864)	0.19	(0.12–0.30)	0.99	(0.66–1.48)
80–100%	26 (790)	0.15	(0.04–0.50)	0.54	(0.33–0.89)
**Age group**					
1–4 years	35 (5661)	1		1	
5–9 years	40 (5244)	1.23	(1.08–1.39)	1.59	(1.46–1.73)
10–14 years	42 (3902)	1.34	(1.12–1.59)	1.76	(1.52–2.03)
**SES tertile**					
Highest	36 (5442)	1		1	
Middle	40 (4926)	1.17	(0.91–1.50)	1.22	(1.00–1.48)
Lowest	41 (4439)	1.21	(0.76–1.92)	1.24	(0.97–1.60)
**Location**					
Bioko Island	22 (9606)	1		1	
Equatorial Guinea	70 (5201)	8.41	(5.85–12.09)	9.55	(6.18–14.76)
**Year of survey**					
2009	38 (6904)	1		1	
2010	39 (7903)	1.06	(0.87–1.30)	1.31	(1.06–1.61)

1 Crude test for linear trend in net condition p = 0.013, OR 0.92 (0.86–0.98). Adjusted test for linear trend in net condition p<0.001, OR 0.89 (0.86–0.93) per increase in condition category.

**Table 5:** Odds ratios (95% CI) of infection with *P. falciparum* among 1–14 year olds in Malawi.

Covariate	% infected (n)	Crude OR	95% CI	Adjusted OR	95% CI
**Protection from net** 1					
Worst and no net	52 (2140)	1		1	
Second worst	53 (288)	1.05	(0.74–1.48)	0.98	(0.75–1.26)
Second best	48 (610)	0.86	(0.70–1.05)	0.84	(0.72–0.98)
Best	45 (475)	0.77	(0.53–1.13)	0.81	(0.56–1.18)
**Site net use**					
<50% people	51 (2417)	1		1	
≥50% people	49 (1096)	0.9	(0.49–1.67)	1.23	(0.64–2.36)
**IRS of the household**					
Unsprayed house	49 (2560)	1		1	
Sprayed within last 12 months	54 (953)	1.25	(0.69–2.25)	1.07	(0.76–1.50)
**Site spray coverage last 12 months**					
0–19.9%	49 (2413)	1		1	
50–79.9%	48 (385)	0.96	(0.48–1.91)	0.42	(0.19–0.94)
80–100%	57 (705)	1.36	(0.66–2.82)	0.7	(0.34–1.43)
**Age group**					
1–4 years	47 (1472)	1		1	
5–9 years	53 (1317)	1.29	(1.10–1.51)	1.33	(1.15–1.54)
10–14 years	52 (724)	1.22	(0.95–1.57)	1.27	(1.03–1.58)
**SES tertile**					
Highest	50 (1471)	1		1	
Middle	52 (1162)	1.06	(0.81–1.38)	1.08	(0.88–1.32)
Lowest	50 (880)	0.98	(0.66–1.45)	1.09	(0.84–1.41)
**Location**					
Non lakeside	34 (1246)	1		1	
Lakeside	59 (2267)	2.8	(1.25–6.27)	3.63	(1.56–8.47)
**Year of survey**					
2009	54 (1881)	1		1	
2010	46 (1632)	0.72	(0.57–0.91)	0.68	(0.47–0.98)

1 Crude test for linear trend in net condition p = 0.083, OR 0.92 (0.84–1.01). Adjusted test for linear trend in net condition p = 0.067, OR 0.93 (0.86–1.01) per increase in condition category.

Protection provided due to living in a community with high IRS coverage (the community IRS effect) was greater than protection offered by living in a sprayed house (the individual IRS effect) (Tables 4 and 5). The adjusted odds ratios show that living in a sprayed house offers some protection (OR = 0.90, 95% CI 0.80–1.01) compared to those living in unsprayed houses in Equatorial Guinea. However, far greater protection was derived from living in an area where IRS coverage was 80% or more, compared to living in an area of less than 20% IRS coverage, irrespective of the spray status of the individual house (OR = 0.54, 95% CI 0.33–0.89). In Malawi there was no reduced risk of infection associated with living in a sprayed house (OR = 1.07, 95% CI 0.76–1.50), however IRS coverage at above 50% offered community protection relative to those living in an area of low IRS coverage (OR = 0.42, 95% CI 0.19–0.94 and OR = 0.7, 95% CI 0.34–1.43 for those living in areas of 50% to <80% coverage and those living in areas of ≥80% coverage respectively).

## Discussion

Our results clearly show that ITNs/LLINs provide protection against malarial infection to those who sleep under them in both Equatorial Guinea and in Malawi and there was no indication of loss of protection from nets that acquired small holes. ITNs/LLINs with large holes were, however, less protective. Untreated nets with no holes offered some protection but untreated nets with holes were not protective. In Equatorial Guinea, where no further LLIN distribution took place during the study period, net ownership declined between 2009 and 2010. Although a decline in net ownership is not uncommon if no continuous distribution mechanism is in place, the steep rate of decline in Bioko may be related to a rapid expansion of new houses on the island (C. Schwabe, unpublished data).

Nets offered personal protection, after adjusting for a number of factors including whether the house was sprayed, the spray coverage of the neighbourhood and the socio-economic status of the household. At the levels of usage of nets seen in both countries, there was no discernable benefit to non users from net usage in the community, confirming the value of nets as being primarily for personal protection in situations where high neighbourhood ITN usage is not achieved. Since no sites had >71% net coverage, conclusions cannot be drawn from this study about the potential community protective effect of ITNs in areas with higher coverage but our findings do not confirm results from previous studies [21], [22] and from modelling [23] which suggests that modest levels of net coverage can achieve community benefits.

In Equatorial Guinea our results confirm the findings of other studies in showing that untreated nets in good condition offer some protection compared to those who do not use nets [6], [7]. In Malawi there were too few untreated nets to observe any potential advantage they may offer relative to not sleeping under a net.

Many ITN/LLIN programs over time suffer from a downward drift in usage and ownership of nets after achieving initial high coverage subsequent to mass distributions. The effect of this reduction in net coverage is further exacerbated by the physical and chemical deterioration in net quality. Our data show how the effectiveness of nets in Equatorial Guinea was impaired as they acquired larger holes. The evidence for this was weaker in Malawi which may be because most nets in Malawi originated from continuous distribution rather than from once off mass distributions as in Equatorial Guinea or it may be simply because of the smaller sample of data that was available from Malawi.

Entomological studies have demonstrated that the effectiveness of ITNs can be compromised by insecticide resistant *An. gambiae* if the nets are holed [9]. In Equatorial Guinea high frequencies of the resistance associated *kdr* mutation have been reported in *An.gambiae* [4], [16], and in Malawi high levels of pyrethroid resistance have recently been detected in *An. funestus* and low levels in *An. arabiensis* (unpublished manuscript in preparation Coleman *et al*). Our data are, however, not suited to investigate whether loss of effectiveness of holed nets is related to the presence of pyrethroid resistance in vectors. Whether the decline in epidemiological effectiveness that we have observed in holed nets is due to insecticide resistance in malaria vectors needs to be investigated. The possibility of operational deterioration of ITN/LLIN effectiveness in areas of insecticide resistance can be countered at least in part by ensuring that nets are in good repair, since untreated nets offer some protection provided they have no holes.

High community coverage of IRS clearly offered the best protection from infection regardless of whether an individual slept in a sprayed or unsprayed house. In Equatorial Guinea community protection was provided only when ≥80% spray coverage was achieved, but there was an additional protective effect provided by living in a sprayed house compared to living in an unsprayed one, regardless of the level of neighbourhood spray coverage. Individual protection from house spraying was not seen in Malawi but there was a community effect at medium spray coverage levels (>50%). The effect at high coverage (≥80%) in Malawi was lower than that at medium coverage. The IRS effects in Malawi may have been influenced by the fact that IRS resources were concentrated where transmission was known to be highest. IRS was primarily conducted at sites near Lake Malawi, whereas in Equatorial Guinea IRS was carried out at all sites in IRS designated provinces (all of Bioko, Litoral and Kie Ntem), regardless of pre-intervention transmission intensity. It is also worth noting that in Malawi pyrethroid insecticide had been used in areas in which recently high levels of pyrethroid resistance has been observed in *An. funestus*, the main vector in the IRS area (Coleman, unpublished data), although the impact of this requires further investigation.

A limitation of this study is that data were obtained from observational malaria indicator surveys rather than randomised trials. As a result there could have been residual confounding in the relationship between infection and quality of vector control that we were unable to adjust for. However, the effects of sub-optimal vector control quality on malarial infection can only be studied in programmatic settings, since random allocation of holed nets or low coverage IRS would be unethical.

Another limitation is the relatively large number of records with missing values in the data. We sought to address this in the analysis using multiple imputation methods which assumes missing data are only related to data we have measured and observed. The results from multiple imputation (not shown) did not differ between a complete case analysis and an imputed analysis suggesting that the assumptions about the missing data were robust [24].

The results of this study have important implications for malaria vector control programmes using ITNs/LLINs or IRS or both. First, malaria control programmes need to put in place monitoring and maintenance strategies to ensure that inadequate nets are replaced since holed and deficient nets are less effective than treated intact ones. Such an approach is currently being planned in Equatorial Guinea using a repair kit which has been developed by the manufacturer to be distributed to householders. Second, IRS must be delivered at high coverage since it offers little or no personal protection and since the community level effect of IRS is only achieved at high coverage. There is little to be gained from low coverage IRS programs.

Our data clearly show how vector control can be of little benefit if it relies on poorly implemented IRS, or on once off LLIN distributions without a replacement and net repair and maintenance strategies. It also underscores the urgent need for manufacturers to produce nets that are physically more durable than most that are currently available.

### Conclusion

Evidence from surveys monitoring operational malaria interventions shows that ITNs with no holes or with small holes give better personal protection against *P.falciparum* than both ITNs with big holes and untreated nets, although the latter provide some protection. Universal ITN coverage strategies need to include effective keep-up and replacement approaches that ensure sustained use of nets in sufficiently good condition. The message for control programs reliant on bed net distribution is therefore not only to seek to provide nets to households that do not have nets, but also to institute strategies for repairing nets, for replacing those that are in non-reparable condition, and to promote proper care and repair of nets to prolong their effective life. Untreated nets that are in a good state of repair will continue to confer some protective effect and are better than no nets at all, irrespective of whether vectors are susceptible to the insecticide or not.

Our results further show that IRS at inadequate coverage provides little or no protection at all, though what constitutes adequate coverage appears to vary by location and transmission characteristics. The message for control programs reliant on IRS is therefore that if IRS is used then it has to be at high coverage levels to be effective.

## Supporting Information

File S1 Generating Socio Economic Status.
(PDF)
