# Supplementary material for: How Much Does Malaria Vector Control Quality Matter: The Epidemiological Impact of Holed Nets and Inadequate Indoor Residual Spraying
Source: PLoS One. 2011 Apr 29;6(4):e19205. doi: 10.1371/journal.pone.0019205 (PMC3084796; doi:10.1371/journal.pone.0019205)
Supplement: File S1 — Generating Socio Economic Status. (PDF) [file pone.0019205.s001.pdf]

Additional file 1: Generating Socio Economic Status

Socio economic status was generated by location (Bioko Island, Equatorial Guinea and Malawi) and year (2009 and 2010) separately. Assets and utilities were examined for missing values in both years and exclusions were made if an asset was missing for a large number of households in both years.

Bioko Island and continental Equatorial Guinea had almost identical questionnaires and hence the asset and utilities listed were almost identical.

For Bioko Island analysis, the only variable with a large number of missing values was whether or not the household owned agricultural land (Table 1.1). The proportion of the variance explained by the first principal component in 2009 was 0.1517 and in 2010 was 0.151. The loadings for each of the assets and utilities are shown in Table 1.2.

There were seven variables (ownership of chickens, cows, goats or sheep, other animals, computer, camera, VCR or DVD) with large numbers of missing values in continental Equatorial Guinea, but no one variable reduced the number of missing values more than others (Table 1.1). They were also variables which could either be associated with high SES or low SES, so all variables were included in the PCA so as not to introduce bias. The proportion of the variance explained by the first principal component in 2009 was 0.1406 and in 2010 was 0.1309. The loadings for each of the assets and utilities are shown in Table 1.2.

In Malawi there were fewer ownership questions in the malaria indicator survey, but there were also very few missing values (Table 1.3). The proportion of the variance explained by the first principal component in 2009 was 0.1503 and in 2010 was 0.1669. Component loadings are shown in Table 1.4. No loadings could be estimated for source of cooking fuel or for ownership of a motorcycle in 2010 because all households used “other cooking fuel source” and no households owned a motorcycle. Similarly in 2009, no households used other water source and hence this component loading could also not be estimated.

Table 1.1: Numbers of missing values for assets and utilities in Equatorial Guinea

	Bioko	Island	Equatorial	Guinea
**Asset or utility**	**Missing n (%)**	**Missing n (%)**	**Missing n (%)**	**Missing n (%)**
	2009	2010	2009	2010
	n=2245	n=2938	n=1742	n=1694
**Household size**	0 (0%)	0 (0%)	0 (0%)	0 (0%)
**Number of rooms**	0 (0%)	0 (0%)	0 (0%)	0 (0%)
**Number of sleeping rooms**	0 (0%)	0 (0%)	0 (0%)	0 (0%)
**Household crowding**	0 (0%)	0 (0%)	0 (0%)	0 (0%)
**Own a radio**	30 (1.3%)	23 (0.8%)	19 (1.1%)	46 (2.7%)
**Own a television**	31 (1.4%)	23 (0.8%)	16 (0.9%)	34 (2%)
**Own a VCR or DVD**	32 (1.4%)	20 (0.7%)	225 (12.9%)	23 (1.4%)
**Own a computer**	42 (1.9%)	35 (1.2%)	225 (12.9%)	22 (1.3%)
**Own a camera**	43 (1.9%)	31 (1.1%)	223 (12.8%)	21 (1.2%)
**Own a telephone**	40 (1.8%)	34 (1.2%)	16 (0.9%)	22 (1.3%)
**Own a clock**	35 (1.6%)	25 (0.9%)	11 (0.6%)	16 (0.9%)
**Own a watch**	56 (2.5%)	20 (0.7%)	12 (0.7%)	19 (1.1%)
**Own chairs**	33 (1.5%)	24 (0.8%)	11 (0.6%)	14 (0.8%)
**Own a sofa**	34 (1.5%)	24 (0.8%)	11 (0.6%)	14 (0.8%)
**Own a table**	34 (1.5%)	24 (0.8%)	12 (0.7%)	17 (1%)
**Own a bed**	32 (1.4%)	24 (0.8%)	11 (0.6%)	13 (0.8%)
**Own an amoire**	44 (2%)	25 (0.9%)	12 (0.7%)	22 (1.3%)
**Own a cabinet**	34 (1.5%)	28 (1%)	13 (0.7%)	20 (1.2%)
**Own fans**	33 (1.5%)	24 (0.8%)	13 (0.7%)	14 (0.8%)
**Own airconditioner**	32 (1.4%)	26 (0.9%)	11 (0.6%)	13 (0.8%)
**Own a refrigerator**	33 (1.5%)	31 (1.1%)	15 (0.9%)	21 (1.2%)
**Own a stove**	32 (1.4%)	24 (0.8%)	13 (0.7%)	16 (0.9%)
**Own a grain grinder**	33 (1.5%)	25 (0.9%)	12 (0.7%)	18 (1.1%)
**Own a washing machine**	32 (1.4%)	26 (0.9%)	12 (0.7%)	20 (1.2%)
**Own a bicycle**	33 (1.5%)	35 (1.2%)	16 (0.9%)	26 (1.5%)
**Own a motorcycle**	33 (1.5%)	31 (1.1%)	14 (0.8%)	25 (1.5%)
**Own a car**	34 (1.5%)	28 (1%)	14 (0.8%)	22 (1.3%)
**Own a motorboat**	35 (1.6%)	29 (1%)	-	-
**Own a sewing machine**	34 (1.5%)	26 (0.9%)	13 (0.7%)	19 (1.1%)
**Own goats or sheep**	58 (2.6%)	26 (0.9%)	24 (1.4%)	89 (5.3%)
**Own chickens**	61 (2.7%)	24 (0.8%)	27 (1.5%)	111 (6.6%)
**Own cows**	46 (2%)	31 (1.1%)	28 (1.6%)	95 (5.6%)
**Own other animals**	81 (3.6%)	31 (1.1%)	28 (1.6%)	125 (7.4%)
**Own agricultural land**	76 (3.4%)	2936 (99.9%)	2 (0.1%)	48 (2.8%)
**Type of toilet**	3 (0.1%)	1 (0%)	0 (0%)	0 (0%)
**Water source**	40 (1.8%)	49 (1.7%)	16 (0.9%)	18 (1.1%)
**Lighting source**	4 (0.2%)	0 (0%)	0 (0%)	0 (0%)
**Type of cooking fuel**	4 (0.2%)	0 (0%)	0 (0%)	0 (0%)
**Rural area**	0 (0%)	0 (0%)	-	-

Table 1.2: Component loadings for assets and utilities in Equatorial Guinea

	Bioko	Island	Equatorial	Guinea
**Asset or utility**	**Loading 2009**	**Loading 2010**	**Loading 2009**	**Loading 2010**
	n=2041	n=2755	n=1459	n=1413
**Household size**	0.0715	0.0447	0.0422	0.0434
**Number of rooms**	0.1581	0.107	0.1139	0.11
**Number of sleeping rooms**	0.0852	0.0824	0.0793	0.0677
**Household crowding**	-0.0756	-0.0884	-0.0589	-0.0559
**Own a radio**	0.1768	0.1908	0.1843	0.1877
**Own a television**	0.2495	0.2469	0.272	0.2742
**Own a VCR or DVD**	0.2345	0.2482	0.2563	0.2768
**Own a computer**	0.1699	0.1805	0.1507	0.1458
**Own a camera**	0.141	0.1605	0.1291	0.1259
**Own a telephone**	0.1976	0.1697	0.1891	0.1978
**Own a clock**	0.2154	0.214	0.205	0.2149
**Own a watch**	0.1884	0.1775	0.1551	0.1826
**Own chairs**	0.0941	0.1321	0.1181	0.1369
**Own a sofa**	0.2028	0.2006	0.2419	0.2282
**Own a table**	0.1223	0.1595	0.1386	0.1471
**Own a bed**	0.0573	0.0638	0.0602	0.0721
**Own an amoire**	0.188	0.202	0.214	0.2104
**Own a cabinet**	0.1982	0.1915	0.1114	0.1072
**Own fans**	0.2387	0.2366	0.2498	0.2138
**Own airconditioner**	0.1211	0.1361	0.1167	0.1153
**Own a refrigerator**	0.2382	0.2513	0.2563	0.2615
**Own a stove**	0.19	0.1833	0.1473	0.1206
**Own a grain grinder**	0.1353	0.1495	0.0321	0.0555
**Own a washing machine**	0.1448	0.1432	0.0733	0.0663
**Own a bicycle**	0.0873	0.0791	0.0586	0.0219
**Own a motorcycle**	0.0366	0.0408	0.0732	0.0583
**Own a car**	0.1578	0.1573	0.1996	0.1799
**Own a motorboat**	0.034	0.0499	-	-
**Own a sewing machine**	0.0308	0.0524	0.0646	0.0333
**Own goats or sheep**	0.0345	0.0164	0.0799	0.0491
**Own chickens**	0.0199	0.0224	0.0255	0.0563
**Own cows**	0.0193	0.0149	0.0056	0.0473
**Own other animals**	0.0329	0.0367	0.0368	0.0145
**Own agricultural land**	-	-	-0.108	-0.074
**Flush toilet**	0.1753	0.1698	0.1516	0.2087
**Pit or VIP toilet**	-0.119	-0.1342	-0.1692	-0.1756
**Other toilet**	-0.075	-0.0629	0.0766	0.0012
**Private piped water**	0.0662	0.0655	0.0695	0.007
**Private well or borehole**	0.0426	0.0232	0.025	0.0618
**Community piped water**	-0.0737	-0.0317	0.0886	0.1038
**Community well or borehole**	0.0386	0.0001	-0.0367	-0.0423

**Other water source**	0.0115	-0.0108	-0.0835	-0.1215
**Electricity for light**	0.1962	-0.2052	-0.2265	-0.216
**Generator for light**	-0.0462	0.1105	0.2287	0.2029
**Other light source**	-0.1921	0.0299	-0.0198	0.0154
**Electricity or gas for cooking**	0.2192	0.2201	0.1657	0.1674
**Kerosene for cooking**	-0.089	-0.1023	0.1028	0.1029
**Other cooking source**	-0.167	-0.1611	-0.2081	-0.2226
**Rural area**	-0.1673	-0.1805	-	-

Table 1.3: Number of missing values for assets and utilities in Malawi

Asset or utility	2009	2010
	Missing n (%)	Missing n (%)
	n=693	n=568
Number of rooms	11 (0.9%)	0 (0%)
Number of sleeping rooms	11 (0.9%)	0 (0%)
Own a radio	11 (0.9%)	0 (0%)
Own a television	11 (0.9%)	0 (0%)
Own a mobile or phone	11 (0.9%)	0 (0%)
Own a bicycle	11 (0.9%)	0 (0%)
Own a motorcycle	11 (0.9%)	0 (0%)
Own a car	11 (0.9%)	0 (0%)
Toilet type	11 (0.9%)	0 (0%)
Water source	11 (0.9%)	0 (0%)
Source of cooking fuel	11 (0.9%)	0 (0%)
Has electricity	11 (0.9%)	0 (0%)

Table 1.4: Component loadings for assets and utilities in Malawi

Variable	2009	2010
	n=682	n=568
Household size	0.2266	0.3635
Number of rooms	0.421	0.4411
Number of sleeping rooms	0.371	0.421
Household crowding	-0.1802	-0.0422
Own a radio	0.2986	0.3418
Own a television	0.2847	0.1894
Own a mobile or phone	0.3446	0.2951
Own a bicycle	0.3066	0.3238
Own a motorcycle	-0.0185	-
Own a car	0.1295	0.0436
Flush toilet	0.0089	-0.0125
Pit or VIP toilet	0.2244	0.1308
Other toilet	-0.2318	-0.1302
Private piped water	0.0454	0.0874
Borehole water source	-0.1312	-0.1707
Community piped water	0.0986	0.1562
Well water	0.1	0.0338
Other water source	-	0.0118
Electricity for cooking	0.0846	-
Other cooking fuel source	-0.0846	-
Has electricity	0.2093	0.2258
